# Is the COBRA‐OS 4 French Aortic Occlusion Device Feasible for Partial REBOA?

**DOI:** 10.1002/wjs.70346

**Published:** 2026-04-08

**Authors:** Adam Power, Asha Parekh, Samuel Jessula, Joao Rezende‐Neto, Laura J. Moore

**Affiliations:** ^1^ Department of Surgery Dalhousie University Halifax Nova Scotia Canada; ^2^ School of Biomedical Engineering Western University London Ontario Canada; ^3^ Trauma and Acute Care General Surgery, Department of Surgery St. Michael's Hospital Toronto Ontario Canada; ^4^ Department of Surgery The University of Texas McGovern Medical School Houston Texas USA

**Keywords:** aortic occlusion, hemorrhagic shock, partial REBOA, REBOA, trauma resuscitation

## Abstract

**Background:**

Noncompressible torso hemorrhage is a leading cause of preventable death. Resuscitative endovascular balloon occlusion of the aorta (REBOA) is used to control hemorrhage but full aortic occlusion is limited by distal ischemia. Partial REBOA (pREBOA) allows some distal perfusion to prolong occlusion, but precise balloon titration is difficult with standard devices. The COBRA‐OS 4 French (Fr) aortic occlusion catheter was evaluated in vitro and in vivo for partial REBOA.

**Methods:**

Bench‐top (in vitro) and porcine (in vivo) models were used. The titration window of the device was evaluated in a non‐hemorrhage porcine model and in a pulsatile aortic model. The ability of the device to maintain a targeted distal aortic pressure (20 mmHg) for an extended period (3 h) was tested in a porcine model of hemorrhagic shock (40% blood volume), with a corresponding in vitro experiment using a pulsatile aortic model.

**Results:**

The COBRA‐OS demonstrated a 3–4 mL linear titration window with 1–1.5 mL deflation volume to reach a target distal aortic pressure of 20 mm Hg in vitro and in vivo. During 3 h of prolonged partial REBOA, the device maintained a stable distal target with a set‐and‐forget strategy (21 +/− 2 mmHg in vitro and 22 +/−7 mmHg in vivo).

**Conclusions:**

The COBRA‐OS 4 Fr device enabled precise, stable partial aortic occlusion in this preclinical model. This is the first demonstration of a 4 Fr REBOA catheter achieving prolonged, controlled partial occlusion in a large‐animal hemorrhagic shock model, supporting the feasibility of this device for pREBOA.

**Level of Evidence:**

Basic Science, Animal study.

## 1 Background

Non‐compressible torso hemorrhage (NCTH) is a leading cause of preventable death in trauma, particularly in military and civilian settings where it accounts for a high proportion of potentially survivable fatalities [1]. Resuscitative endovascular balloon occlusion of the aorta (REBOA) has emerged as a method to temporize hemorrhage by inflating a balloon in the aorta to interrupt distal blood flow, thereby gaining time for surgical hemostasis [2]. REBOA can significantly raise central blood pressure and improve perfusion to the heart and brain during hemorrhagic shock [3]. However, complete aortic occlusion also causes ischemia in all tissues distal to the balloon, leading to severe reperfusion injury once flow is restored. Consequently, fully occlusive REBOA in the supra‐diaphragmatic aorta (Zone 1) is conventionally limited to 30 min to avoid catastrophic ischemic complications [4]. Although this technique is beneficial for many patients, the time limit poses a major challenge: definitive hemorrhage control often cannot be achieved within such a short window, especially in scenarios with prolonged transport or complex surgical access.

Partial REBOA (pREBOA) has been proposed to address this problem. In pREBOA, the balloon is deliberately under‐inflated or gradually deflated to allow some distal aortic perfusion while maintaining proximal perfusion and hemorrhage control [5]. By creating a controlled degree of aortic flow, effectively a “permissive hypotension” below the balloon, pREBOA seeks to balance bleeding control with reduced ischemic burden. Preclinical studies have demonstrated that partial aortic occlusion can substantially prolong tolerable occlusion times and attenuate metabolic derangements compared to complete occlusion [6]. For example, a recent swine study achieved Zone 1 partial occlusion for up to 4 h with only mild, surgically manageable ischemic injury in distal organs [7].

Despite its promise, pREBOA can be technically challenging. Most REBOA catheters in use have compliant balloon segments designed to prevent damage to the aortic wall on inflation [8]. While beneficial for avoiding aortic injury, compliant balloons can be difficult to titrate during deflation; a small volume change can lead to a sudden large increase in distal flow, causing a precipitous drop in proximal pressure [9]. In practice, some providers have found it difficult to maintain a stable partial occlusion using standard balloons, and abrupt deflation can result in significant hypotension. These limitations spurred the development of new REBOA devices aimed at facilitating partial occlusion [10].

The COBRA‐OS (Control Of Bleeding, Resuscitation, Arterial Occlusion System) (London, Ontario, Canada) is a REBOA device that may offer advantages for pREBOA. It is an aortic occlusion catheter with an extremely low profile (inserted through a 4 Fr sheath) and a fully compliant balloon. Initial testing demonstrated that despite its compliant balloon, the COBRA‐OS could be titrated to allow partial flow [11]. However, the study was limited to short‐duration trials without hemorrhage. Despite anecdotal uses of this device for pREBOA, it remains unknown whether the COBRA‐OS can maintain a stable partial occlusion during active hemorrhagic shock and for clinically relevant durations.

The aim of this study was to determine whether the COBRA‐OS device is titratable for pREBOA and whether it can safely perform and sustain pREBOA in a severe hemorrhage scenario.

## 2 Methods

*Experimental Groups*: Four experimental groups were conducted (Table 1). Groups 1 and 2 examined the COBRA‐OS in *incremental* partial occlusion trials (in vitro and in vivo without hemorrhage), to characterize the titration window of the device for partial occlusion. Groups 3 and 4 examined *sustained* partial occlusion in hemorrhage conditions (in vitro and in vivo), to test prolonged safety and performance. The target distal pressure of 20 mmHg was selected to provide limited but non‐zero perfusion, based on prior pREBOA studies achieving approximately 20 mmHg distal systolic pressure as a partial occlusion endpoint [7].

- *Group 1: In Vitro Incremental pREBOA*. A closed‐loop pulsatile flow model (United Biologics, CA, USA) of the thoracic aorta was used (simulated 21 mm diameter aorta). Starting from full occlusion, the COBRA‐OS balloon was then incrementally deflated in small volume steps (0.2 mL every 30 s) until full aortic flow was restored (no contact between balloon and wall and equalization of pressures above and below the balloon). At each step, the proximal pressure (above the balloon) and distal pressure (below the balloon) were recorded. This protocol was performed three times, and the results were averaged.
- *Group 2: In Vivo Incremental pREBOA (Non‐Hemorrhagic)*. In three anaesthetized swine (no hemorrhage), the COBRA‐OS was placed in aortic Zone 1 (mid‐descending thoracic aorta distal to the left subclavian) and inflated to full occlusion. Then, similar to Group 1, the balloon was gradually deflated in small volume increments (0.2 mL every 30 s) until the distal pressure equilibrated with the proximal, indicating near‐full restoration of flow.
- *Group 3: In Vitro Sustained pREBOA (Simulated Hemorrhage)*. The COBRA‐OS was deployed in the closed‐loop pulsatile flow model, and initial flow/pressure were set to mimic a shocked state (50 mmHg simulated MAP). The balloon was inflated to occlusion and then partially deflated to reach 20 mmHg distal pressure. This partial occlusion scenario was maintained for 3 h while recording proximal and distal pressures. This protocol was performed three times, and the results were averaged.
- *Group 4: In Vivo Sustained pREBOA (Hemorrhagic Shock)*. Three swine were subjected to a controlled hemorrhage and prolonged pREBOA. The hemorrhage protocol was adapted from a prior hemorrhagic swine model for pREBOA [7]. Animals had 40% of their estimated blood volume rapidly removed via a femoral venous line over 20 min, inducing Class IV hemorrhagic shock, which resulted in tachycardia and hypotension. The animal was maintained in this shocked, hypotensive state for 60 min with no intervention (mimicking a delayed prehospital phase). After 1 h of shock, the COBRA‐OS was inflated to full occlusion initially and then carefully deflated until the distal MAP stabilized at 20 mmHg and pREBOA was maintained for 3 h. After 3 h of partial occlusion, the shed autologous blood was transfused back intravenously over 10 min while the balloon was simultaneously slowly deflated over this time. The animal was then observed for an additional hour under anesthesia with standard critical care.

**TABLE 1:** Summary of experimental groups.

Group	Setting	Hemorrhage condition	Partial REBOA protocol
1. In vitro—Incremental pREBOA	Pulsatile flow bench model (simulated aorta)	No hemorrhage	Inflate balloon to full occlusion, then stepwise deflate (0.2 mL steps) to incrementally restore flow
2. In vivo—Incremental pREBOA	Anaesthetized swine (zone 1 aortic placement)	No hemorrhage	Inflate balloon to full occlusion, then gradually deflate in small increments (0.2 mL steps) under continuous hemodynamic monitoring to observe controlled distal reperfusion
3. In vitro—Sustained pREBOA	Pulsatile flow bench model (simulated aorta)	Simulated hemorrhage	Inflate balloon to occlusion in circuit, then partial occlusion at 20 mmHg distal pressure maintained for 3 h (no adjustments after initial titration)
4. In vivo—Sustained pREBOA	Anaesthetized swine (zone 1)	40% hemorrhage, then 60 min shock	Inflate balloon to occlusion after hemorrhagic shock; then partial occlusion at 20 mmHg distal MAP maintained for 3 h (no adjustments after initial titration)

*Animal Preparation*: This animal portion of the study was approved by the institutional Animal Care Committee, and all procedures conformed to ARRIVE guidelines. Female domestic swine (Sus scrofa, Landrace‐Yorkshire cross) weighing approximately 43–48 kg were used for in vivo experiments. Following anesthesia induction, general anesthesia was maintained with isoflurane (2%–5% in oxygen) and intermittent positive‐pressure ventilation. Each pig was instrumented with vascular access lines: a 7 Fr sheath in the left common carotid artery for proximal aortic pressure monitoring, a 7 Fr sheath in the left internal jugular vein and left common femoral vein for fluid resuscitation and medications, a 7 Fr sheath in the left femoral artery for distal pressure monitoring, and a 4 Fr arterial sheath in the right femoral artery for COBRA‐OS catheter insertion. Continuous invasive blood pressure monitoring (proximal and distal) was recorded via a data acquisition system.

*Data Collection and Analysis*: For each group, key hemodynamic variables were recorded and summarized. In groups 1 and 2, the primary outcome was the titration range of the COBRA‐OS balloon (change in balloon volume between full occlusion and the point of significant distal reperfusion) and the corresponding proximal versus distal MAP relationship. In groups 3 and 4, the primary outcome was the ability to maintain the target distal MAP (20 mmHg) for 3 h, the proximal hemodynamics during that period, and markers of ischemia reperfusion after balloon deflation. We also qualitatively noted any major events (balloon rupture, migration, or loss of occlusion) and any physiological responses (arrhythmias). Given the exploratory nature of this study, no formal statistical hypothesis testing was applied. Descriptive statistics (mean +/− SD) were reported for repeated measures. The sample size of 3 animals per in vivo subgroup was chosen based on prior feasibility studies in this domain and ethical considerations to use the minimum number of animals necessary. All animals were included unless they met predefined exclusion criteria and no animals were ultimately excluded. Generative artificial intelligence (ChatGPT) was used in the editing process of this paper to improve readability.

## 3 Results

**FIGURE 1:**
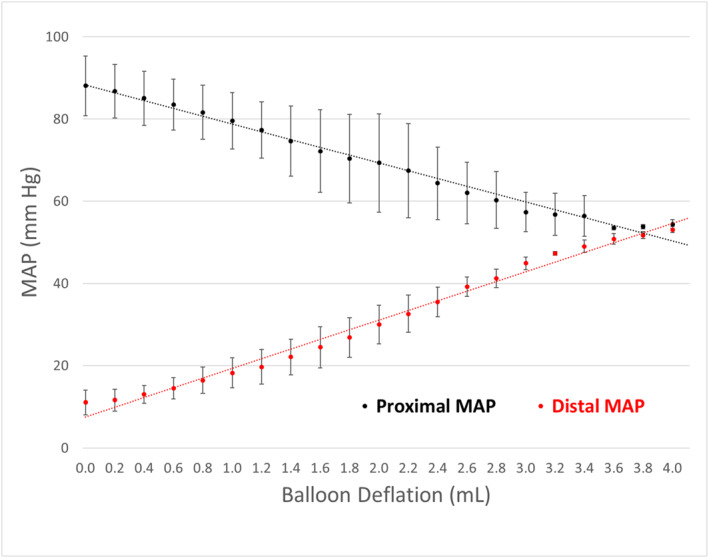
Graph of the mean proximal and distal mean arterial pressures (MAPs) produced by incremental balloon deflation of the COBRA‐OS in vivo.

**FIGURE 2:**
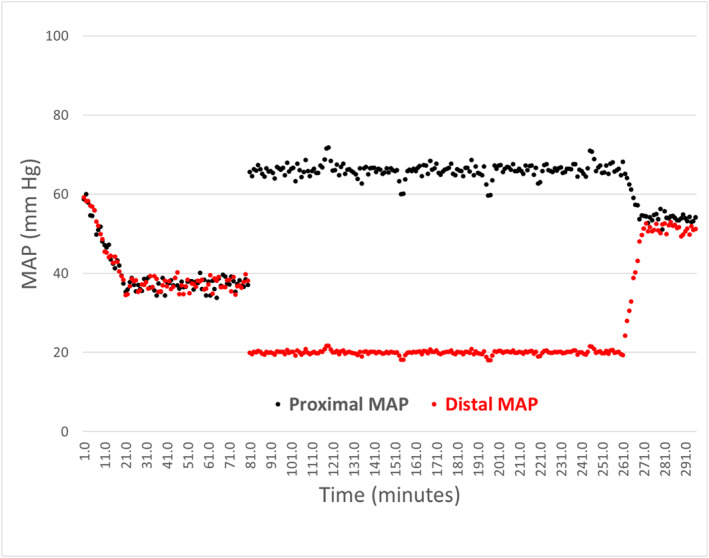
Hemodynamic response to hemorrhage and prolonged partial REBOA (pREBOA) using the COBRA‐OS.

1. *In Vitro Incremental Partial Occlusion*: The COBRA‐OS demonstrated a clear titration window during bench‐top deflation testing. Once the distal MAP started to increase and subsequently changed from 0 to 20 mmHg, a 1.2 +/− 0.1 mL deflation range was observed. Within this deflation interval, the COBRA‐OS allowed a controlled transition from total occlusion to partial flow. Beyond this window (greater deflation), distal pressure rose linearly toward near equality with proximal pressure. In practical terms, the COBRA‐OS balloon provided a 3.3 +/− 0.1 mL volume range over which it could modulate between full and partial occlusion in vitro. The balloon maintained its position and integrity throughout. There were no leaks or failures during the repeated inflations/deflations.
2. *In Vivo Incremental Partial Occlusion (No Hemorrhage)*: With the COBRA‐OS deployed in Zone 1, full inflation raised proximal MAP from 64 +/−12 mmHg to 88 +/− 7 mmHg. A titration window deflation balloon volume change of 1.1 mL +/− 0.2 mL for 20 mmHg distal MAP and 3.7 ± 0.3 mL for equalization of proximal and distal pressures was documented. Figure 1 shows this relationship. Notably, partial deflation to a distal MAP of 20 mmHg preserved a significant proximal‐distal pressure gradient, with a proximal MAP of greater than 75 mmHg from baseline 64 +/−12 mmHg. The animals experienced no arrhythmias, cardiac events, or hemodynamic instability during balloon manipulation.
3. *In Vitro 3‐Hour pREBOA Simulation*: The COBRA‐OS devices maintained the target distal pressure without operator intervention. The distal pressure was 21 +/− 2 mmHg for the 3 h timeframe while the proximal pressure in the circuit was 64 +/− 3 mmHg during partial occlusion. The COBRA‐OS maintained its balloon shape during the intervention time with no balloon leaks or failures.
4. *In Vivo 3‐Hour pREBOA (Hemorrhagic Shock)*: All three animals subjected to hemorrhage and prolonged pREBOA survived the 3 h occlusion period and subsequent reperfusion phase. After the 40% blood volume hemorrhage (20 min), pigs were in profound shock (pre‐REBOA proximal MAP 37 mmHg +/−8 mmHg, heart rate 136 +/−17/min) and stayed in shock for 60 min. Inflation of the COBRA‐OS balloon increased proximal MAP to 66 +/−7 mmHg, while distal MAP fell to < 5 mmHg (complete occlusion). The balloon was then deflated slowly over 0.2 mL increments and adjusted over 15 min while distal MAP stabilized at 20 mmHg. The mean deflation volume from full occlusion was 1.4 mL +/− 0.3 mL. Once set, the balloon position and volume were held constant for 3 h. During this period, the ratio of the proximal MAP to distal MAP of the animals remained constant even during periods of relative hypertension or hypotension (3.3 +/− 0.1). The proximal MAP during the 3 h period was mean 59 +/−11 mmHg with partial occlusion, and distal MAP was mean 22 +/−7 mmHg (Figure 2).
  Physiologically, there were no episodes of severe hypertension or severe hypotension during partial occlusion. Urine output was scant (as expected during shock) but detectable in all animals, indicating that some renal blood flow was preserved. Throughout the occlusion, no arrhythmias or cardiac ischemic signs were observed. There were also no balloon‐related issues.
  At the conclusion of 3 h, proximal MAP decreased to mean 54 mmHg +/− 8 mm Hg after full deflation, and distal MAP equilibrated to 51 mmHg +/− 6 mm Hg. Over the final hour of intensive care, hemodynamics gradually normalized in all animals (MAP 62 +/− 9 mm Hg, heart rate 111 +/− 16/min). One animal had a brief run of ventricular tachycardia but otherwise, no other arrhythmias were noted.

## 4 Discussion

In this study, we demonstrated that the COBRA‐OS, a 4 Fr compliant balloon aortic occlusion catheter, could achieve effective and sustained partial aortic occlusion in both bench‐top and in vivo hemorrhage and non‐hemorrhage models. To our knowledge, this is the first report of a 4 Fr REBOA device successfully maintaining a targeted low distal perfusion pressure for hours in a large‐animal hemorrhagic shock scenario. These findings highlight the potential of the COBRA‐OS as a tool for pREBOA.

**FIGURE 3:**
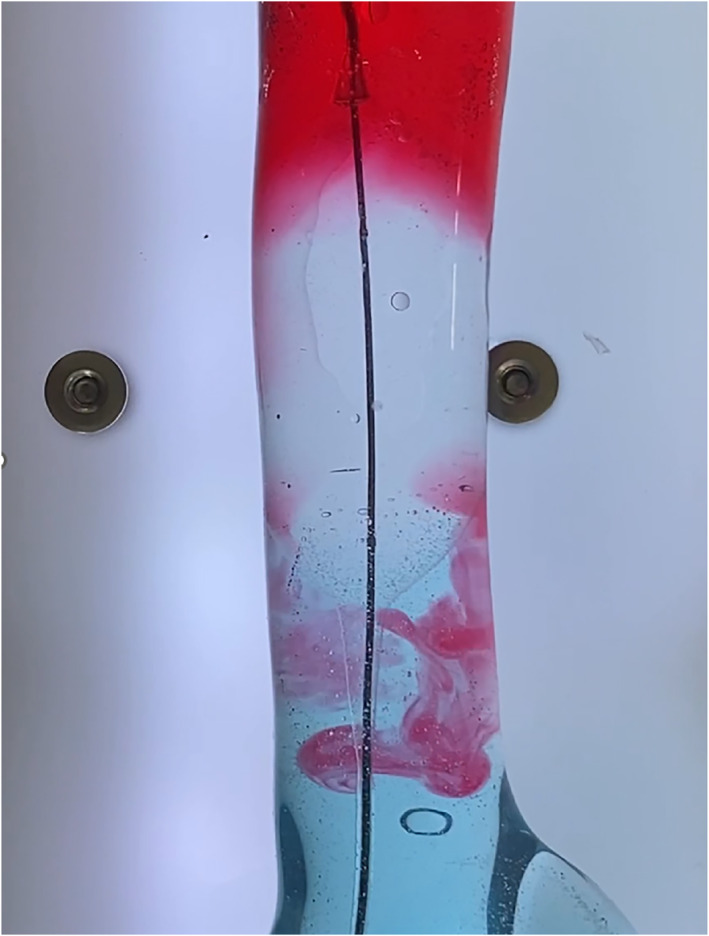
Image from a partial REBOA video of the COBRA‐OS in the benchtop model showing the proximal fluid (red) slowly mixing with the distal fluid (blue).

The balloon of the COBRA‐OS was engineered to have an offset shape to counteract the “tear‐drop” shape that normally forms when deflating the balloon of older generation REBOA devices [12]. This deflation tear‐drop effect on the aortic wall combined with the inability to remove small aliquots easily from the balloon causes most spherical compliant balloons to act in an “all‐or‐nothing” fashion. The COBRA‐OS balloon has a shape and mechanical properties that allow it to maintain its overall shape throughout inflation and deflation cycles. Additionally, the forced slow deflation of the device due to the smaller deflation flow channel facilitates removing small aliquots out of the balloon and therefore can prevent abrupt changes in flow (Figure 3, Video 1). In practical terms, the COBRA‐OS provided a sizable volume window (3–4 mL) over which pressure could be titrated, allowing a steady distal MAP (20 mmHg) to be held for 3 h with minimal intervention. Importantly, the transition from full occlusion to partial was smooth and did not cause hemodynamic instability; there was no precipitous drop in proximal pressure when we eased the balloon, in contrast to the “central hypotension” that can occur when deflating traditional compliant balloons.

**Video 1:** Video of COBRA‐OS transitioning from full occlusion to partial occlusion in a pulsatile flow model for demonstration purposes. To view this video in the full‐text HTML version of the article, please visit https://onlinelibrary.wiley.com/doi/10.1002/wjs.70346.

Our findings with the COBRA‐OS illustrate that pREBOA is a technique and not necessarily a property unique to one catheter. We successfully performed pREBOA by applying the principles of controlled inflation/deflation. Beyond our study, the device historically used for REBOA in trauma was the Cook Coda (Cook Medical, IN, USA) balloon catheter. In some early cases practitioners would intermittently deflate the Coda balloon to allow periodic reperfusion (an early form of intermittent or partial REBOA) [13]. While not designed specifically for partial use, these experiences showed that skilled operators could manually create a partial occlusion scenario with existing tools. Recently, a specifically designed pREBOA device, the pREBOA‐PRO (Prytime Medical, TX, USA), has been investigated for pREBOA purposes clinically [14].

In clinical practice, pREBOA with the COBRA‐OS can be performed by first transducing the sidearm of the supplied 4 Fr sheath for distal MAP measurements. When the device is introduced into the sheath, the waveform is blunted but the MAP reading remains accurate. If the user wants to visualize the full waveform with the deflated device in place, then a standard 5 Fr sheath can be used with the sidearm transduced. With full aortic occlusion, the MAP is close to 0 and no pulsatile waveform is seen. The stopcock can then be opened (the balloon deflates slowly on its own without withdrawal on the syringe being necessary) until a pulsatile wave form first appears and then the stopcock is closed. A preferred distal MAP can then be adjusted by removing 0.2–0.4 mL aliquots until the target distal MAP is achieved.

The encouraging outcomes in the 3 h hemorrhagic pREBOA animals suggests that pREBOA can extend the safe occlusion time. In prior complete REBOA studies, 60 min of Zone 1 occlusion often resulted in severe lactic acidosis, organ damage, and high mortality after release. Here, with partial occlusion, our animals remained viable and responsive after 180 min. This aligns with recent work by Ho et al. (2023), who showed 100% survival in pigs with 120 min of partial Zone 1 occlusion using the pREBOA‐PRO, and even tolerable (if suboptimal) outcomes with 240 min partial occlusion [7]. Taken together, it appears pREBOA, whether via COBRA‐OS or other means, can safely bridge multiple hours of hemorrhagic shock. This could be lifesaving in scenarios such as long battlefield evacuation, remote rural trauma, or multi‐casualty events where definitive care is delayed.

While our study focused on pREBOA, the concept of intermittent REBOA (iREBOA), periodically inflating and deflating to allow reperfusion, is another strategy to extend occlusion time [15]. Intermittent REBOA, however, may cause significant blood pressure oscillations and might destabilize patients. Thus, some users of the COBRA‐OS have adopted an “Intermittent Partial REBOA” approach, switching between complete and partial REBOA without ever deflating the balloon completely, until REBOA is no longer needed.

This study is a proof‐of‐concept with a small sample size. It was not randomized or controlled against full occlusion (because the deleterious effects of full 3 h occlusion are already well known). Instead, our focus was demonstrating feasibility and safety of pREBOA with the COBRA‐OS. Another limitation is that we did not measure certain parameters (detailed chemistry, pathology, and histology) that would have given a fuller picture of reperfusion injury. The decision to omit these parameters was due to the expectation (from prior work) that gross physiologic derangements would be a clear indicator of severe ischemia. Additionally, while our protocol simulated a prolonged prehospital scenario, real‐world variability (ongoing bleeding rate, need for additional interventions, patient comorbidities) could influence outcomes. Finally, we attempted to make the model clinically realistic, but no animal model is perfect.

## 5 Conclusion

The COBRA‐OS 4 Fr aortic occlusion device proved capable of effective and stable pREBOA in our study using in vitro and in vivo, hemorrhagic and non‐hemorrhagic models. The device allowed precise titration of balloon deflation and was able to maintain a low distal perfusion pressure for 3 h. These results suggest that this fully compliant, low‐profile balloon catheter can be utilized for partial aortic occlusion.

## Author Contributions

**Adam Power:** conceptualization, investigation, funding acquisition, writing – original draft, methodology, validation, visualization, writing – review and editing, formal analysis, project administration, data curation, supervision, resource. **Asha Parekh:** conceptualization, data curation, formal analysis, funding acquisition, investigation, methodology, validation, writing – review and editing. **Samuel Jessula:** validation, writing – review and editing. **Joao Rezende‐Neto:** data curation, validation, writing – review and editing. **Laura J. Moore:** validation, writing – review and editing.

## Funding

The study was supported by Front Line Medical Technologies Inc.

## Conflicts of Interest

A Power and A Parekh are co‐founders and have an equity stake in Front Line Medical Technologies Inc. LJM is Chair of the Scientific Advisory board, has an equity stake, and receives consulting fees from Front Line Medical Technologies Inc. JRN is co‐founder and has an equity stake in InventoRR MD Inc. and AbClo Surgical; also has shares in Revolve Surgical and Fluid AI Medical. Front Line Medical Technologies paid the expenses for this animal study.

## Data Availability

The data that support the findings of this study are available on request from the corresponding author. The data are not publicly available due to privacy or ethical restrictions.
